# *PADI4* Haplotypes Contribute to mRNA Expression, the Enzymatic Activity of Peptidyl Arginine Deaminase and Rheumatoid Arthritis Risk in Patients from Western Mexico

**DOI:** 10.3390/cimb44090293

**Published:** 2022-09-16

**Authors:** Mónica Guadalupe Matuz-Flores, Jesús Alfredo Rosas-Rodríguez, Orlando Tortoledo-Ortiz, Salvador Muñoz-Barrios, Gloria Esther Martínez-Bonilla, Jorge Hernández-Bello, Christian Johana Baños-Hernández, Cesar Pacheco-Tena, Gabriela Athziri Sánchez-Zuno, Beatriz Panduro-Espinoza, José Francisco Muñoz-Valle

**Affiliations:** 1 Instituto de Investigación en Ciencias Biomédicas, Centro Universitario de Ciencias de la Salud, Universidad de Guadalajara, Guadalajara 44340, Jalisco, Mexico; 2 Departamento de Ciencias Químico Biológicas y Agropecuarias, Universidad de Sonora Unidad Regional Sur, Navojoa 85880, Sonora, Mexico; 3 Centro de Investigación en Alimentación y Desarrollo A.C., Coordinación de Nutrición, Lab. de Cromatografía, Hermosillo 83304, Sonora, Mexico; 4 Unidad Académica de Ciencias Naturales, Universidad Autónoma de Guerrero, Chilpancingo 39086, Guerrero, Mexico; 5 Servicio de Reumatología, Hospital Civil de Guadalajara Fray Antonio Alcalde, Guadalajara 44280, Jalisco, México; 6 Facultad de Medicina y Ciencias Biomédicas, Universidad Autónoma de Chihuahua, Chihuahua C.P. 31109, Chih., Mexico

**Keywords:** ACPA, *PADI4* gene, *PADI4* expression, PAD4 activity, Rheumatoid arthritis

## Abstract

Citrullination is catalyzed by the peptidyl arginine deiminase 4 (PAD4) enzyme, encoded by the *PADI4* gene. Increased PAD4 activity promotes the onset and progression of rheumatoid arthritis (RA). This study aimed to evaluate the association of *PADI4* haplotypes with RA risk, mRNA expression, and the PAD4 activity in patients with RA from Mexico. Methodology: 100 RA patients and 100 control subjects (CS) were included. Genotyping was performed by PCR-RFLP method, *PADI4* mRNA expression was quantified by real-time PCR, the contribution of *PADI4* alleles (*PADI4_89* G>A, *PADI4_90* T>C, and *PADI4_92* G>C) to mRNA expression by the ASTQ method, and PAD4 activity by HPLC. Also, the anti-CCP and anti-PADI4 antibodies were quantified by ELISA. Results: The three *PADI4* polymorphisms were associated with RA susceptibility (OR = 1.72, *p* = 0.005; OR = 1.62; *p* = 0.014; OR = 1.69; *p* = 0.009; respectively). The 89G, 90T, and 92G alleles have a higher relative contribution to *PADI4* mRNA expression from RA patients than 89A, 90C, and 92C alleles in RA patients. Moreover, the GTG/GTG haplotype was associated with RA susceptibility (OR = 2.86; *p* = 0.024). The GTG haplotype was associated with higher *PADI4* mRNA expression (*p* = 0.04) and higher PAD4 enzymatic activity (*p* = 0.007) in RA patients. Conclusions: The evaluated polymorphisms contribute to *PADI4* mRNA expression and the enzymatic activity of PAD4 in leukocytes. Therefore, the GTG haplotype is a genetic risk factor for RA in western Mexico, and is associated with increased *PADI4* mRNA expression and higher PAD4 activity in these patients.

## 1. Introduction

Rheumatoid arthritis (RA) is an autoimmune, inflammatory, chronic, and systemic disease characterized by dysregulation of cytokine production and the presence of antibodies against citrullinated proteins (ACPA) [1].

One of the most relevant molecules in the development of this disease is the enzyme peptidyl arginine deaminase type IV (PAD4). The PAD4 enzyme catalyzes the conversion of arginine to citrulline residues. In humans, five isoforms of PAD have been described, PAD1, PAD2, PAD3, PAD4, and PAD6, distributed in many cells and tissues. Among these, PAD4 is expressed primarily on neutrophils, eosinophils, and monocytes [2,3].

High activity of PAD4 has been associated with the appearance and progression of RA because it promotes neutrophils recruited to the site of inflammation and the formation of neoantigens, and the hypercitrullination of vimentin, fibrinogen, type II collagen, and α-enolase [4,5,6], which are the primary targets of the autoantibodies in RA.

Also, PAD participates in biological processes such as the formation of extracellular neutrophil traps (NETs), which contain nuclear material, granular enzymes, and cytoplasmic proteins that can be a source of autoantigens during NETosis in autoimmune diseases [7].

The PAD4 enzyme is encoded by the *PADI4* gene, which has three single nucleotide polymorphisms (SNPs) that generate amino acid substitutions (*PADI4_89* G>A [rs11203366], *PADI4_90* T>C [rs11203367], and *PADI4_92* G>C [rs874881]): S55G, A82V, and A112G, respectively. These polymorphisms also generate a conformational change in the enzyme [8]. The conformational change caused by these SNPs can affect the interaction of the enzyme with the substrates [9].

The 3 *PADI4* SNPs are in strong linkage disequilibrium, and form haplotypes. The GTG haplotype has been associated with RA, higher ACPA levels, and mRNA expression in several populations such as Japanese [8,10], Korean [11], North American [12], and western and southern Mexico [13,14]. In addition, the activity of the PAD enzyme on synovial fluid has been shown, and it correlates with ACPA positivity, disease activity, and inflammatory mediator concentrations [15,16]. However, the effects of the GTG haplotype on the activity of the PAD4 enzyme in polymorphonuclear cells (PMN) and peripheral blood mononuclear cells (PBMC) of RA patients have been poorly explored. Therefore, this study aimed to identify the relationship between *PADI4* gene haplotypes with RA risk, mRNA expression in PBMC, and PAD4 activity in the leukocytes (PMN and PBMC) of Mexican patients.

## 2. Materials and Methods

### 2.1. RA Patients and Control Subjects

One hundred RA patients from western Mexico were included, classified according to the ACR/EULAR 2010. They were recruited from the rheumatology service at the Hospital Civil “Fray Antonio Alcalde”, Guadalajara, Jalisco. Their clinical activity and functional disabilities were evaluated by the DAS28 score (disease activity score) [17], and a visual analog pain scale HAQ-DI (Health Assessment Questionnaire Disability Index), respectively [18,19]. Moreover, one hundred CS classified by self-report were included, which were recruited from the general population. Only Mexican Mestizo unrelated subjects with ancestry from western Mexico (Jalisco, Nayarit, Colima, and Michoacan states) since three previous generations were enrolled in the study, to prevent population heterogeneity. The ethics committee of the University of Guadalajara approved this study (CI-05718), which was conducted following the ethical principles established in the Declaration of Helsinki (64th General Assembly, Fortaleza, Brazil, October 2013) and the Official Mexican Standard NOM-087-ECOL-SSA1-2002, Environmental protection, Environmental health, Hazardous biological-infectious waste—Classification and handling specifications. The subjects who participated in this study each signed an informed consent letter.

### 2.2. Laboratory Assessment and Quantification of Qutoantibodies

Peripheral blood samples were obtained from all subjects included in the study. Serum was isolated using standard protocols, aliquoted, and stored at −80 °C until use. The clinical parameters, C-reactive protein (CRP) and rheumatoid factor (RF), were determined by turbidimetry (BS120 Chemestry Analyzer, 2, Shenzhen, China) using an assay based on latex microparticles (COD31029; BioSystems, Barcelona, Spain), and with the RF latex assay (COD31030; BioSystems, Barcelona, Spain), respectively, according the manufacturer’s instructions. Individuals with RF values > 30 IU/mL were considered positive. The determination of ESR was carried out with the Wintrobe method. The quantification of antibodies against cyclic citrullinated peptides (anti-CCP) was carried out using an enzyme-linked immunosorbent assay (ELISA) (Axis-Shield Diagnostics, Scotland, UK); serum values > 5 U/mL were considered positive. An ELISA based on the determination of any anti-PADI4 immunoglobulin isotype (IgG, IgM, and IgA) was performed according to the manufacturer’s recommendations (Cayman Chemical Co, Ann Arbor, MI, USA) for the serum anti-PADI4 antibodies quantification.

### 2.3. PADI4 SNPs Genotyping

Three exonic *PADI4* SNPs were genotyped using the PCR-RFLP method. The sets of primers were those reported by Guzman-Guzman et al. [11]. Since the *PADI4* 89G>A and 90T>C SNPs are in proximate sequences, a single PCR reaction was performed to amplify both polymorphic fragments. Reactions were carried out in a final volume of 25 µL containing 0.12 µM of each primer, 100 ng of gDNA, 2.5 U of *Taq* DNA polymerase (InvitrogenTM life technologies, Carlsbad, CA, USA), 1X enzyme supplied buffer, 4.5 mM MgCl_2_, 1.4 M Betaine (SIGMA Life Science, Misuri, USA), and 0.1 mM of dNTPs (InvitrogenTM life technologies, CA, USA). The cycling conditions were the following: initial denaturation at 94 °C for 3 min (min); followed by 30 cycles of 30 s (s) at 94 °C, 30 s at 62 °C, and 30 s at 72 °C; each with an ending extension step of 1 m at 72 °C. The resultant 221 bp fragment was afterward digested in separate reactions with the *BtgI* enzyme or with the *MscI* enzyme (New England, BioLabs, Inc., Watertown, MA, USA), to identify the *PADI4* 89G>A and 90T>C polymorphisms, respectively. The digested products were then electrophoresed on a 6% polyacrylamide gel (29 acrylamide: 1 bisacrylamide) and stained with 2% AgNO3. In the *PADI4* 89G>A polymorphism, the G allele was identified by two fragments (144 and 77 bp), and the A allele was recognized as a 221 bp fragment; whereas in the *PADI4* 90T>C polymorphism, the T allele was represented by two fragments (165 and 56 bp), a single 221 bp fragment represented the C allele. For the *PADI4* 92G>C genotyping, PCR was performed in a final volume of 15 µL containing 0.2 µM of each primer, 100 ng of gDNA, 2.5 U *Taq* DNA polymerase (Invitrogen™ life technologies, USA), 1X enzyme supplied buffer, 2.5 mM MgCl_2_, and 0.2 mM dNTPs (InvitrogenTM life technologies, USA), under the following conditions: initial denaturation at 94 °C for 3 min followed by 35 cycles of 30 s at 94 °C, 30 s at 56 °C, and 30 s at 72 °C; then, a final extension of 1 min at 72 °C. The PCR product resulted in a 106 bp fragment subsequently digested with the *MspI* restriction endonuclease (New England BioLabs, Inc.). The products were then electrophoresed on a 6% polyacrylamide gel and stained with AgNO3. The G allele was represented as two fragments (59 and 47 bp), while the C allele resulted in a single fragment of 106 bp. A 50 bp molecular weight standard (Invitrogen™ life technologies, CA, USA) and the corresponding positive/negative controls for each *PADI4* SNP were loaded in all polyacrylamide gels.

### 2.4. RNA Isolation and cDNA Synthesis

Total RNA was extracted immediately from 5 mL of EDTA—treated peripheral blood of RA patients and controls. Total leucocytes were isolated using 5% dextran solution (Sigma-Aldrich, St. Louis, MO, USA), and the RNA was obtained using TRIzol reagent (InvitrogenTM life technologies, CA, USA), according to the manufacturer’s instructions. The RNA was analyzed on a NanoDrop 2000 spectrophotometer (Thermo Scientific, MA, USA) by measuring the optical density (OD) at 260 nm for the quantity and the OD260/280 ratio for the quality. Before cDNA synthesis, RNA samples were treated with DNAse I (InvitrogenTM Life Technologies, CA, USA). 1 µg of total RNA was subjected to reverse transcription using oligo(dT) and M-MLV reverse transcriptase, as indicated by the manufacturer (Promega, Madison, WI, USA).

### 2.5. Quantification of PADI4 Allelic Contribution to the mRNA Expression of PADI4

In the samples identified as heterozygous for the polymorphisms 89G>A, 90T>C, and 92G>C of the *PADI4* gene, the allelic contribution to mRNA expression of *PADI4* was determined by the allele-specific transcript quantification (ASTQ) method reported by Kaijzel et al. [14]. It allowed discriminating the mRNA expression of two alleles through quantifying fragments digested by restriction enzymes and the ratio of bands representing the allelic expression that reflects the relationship between the respective precursor transcripts. From 100 ng of cDNA, we proceeded to PCR amplification and subsequent digestion with the respective restriction enzymes (*Btg I*, for 89G>A; *Msc I* for 90T>C; and *Msp I* for 92G>C), using 10 U for each experiment. The densitometry of the bands corresponding to the products digested with the respective restriction enzymes was performed using ImageJ software (National Institutes of Health, New York, NY, USA) from 3% agarose gels (Vivantis Technologies, Ehsan, Malaysia) stained with GelRed 1X (Biotium Inc, Fremont, CA, USA). The intensity of the band was automatically determined in RUA.

### 2.6. Quantitative Real-Time PCR for PADI4 Expression Quantitative

The quantification of *PADI4* mRNA was conducted by real-time PCR using TaqMan^®^ probes (Applied Biosystems, Waltham, MA, USA) as a fluorescent system for both the interest gene *PADI4* (ID Hs00202612 m1) and the reference gene glyceraldehyde 3-phosphate dehydrogenase (*GAPDH*) (ID Hs03929097 g1). All samples were analyzed in duplicate using the conditions indicated in the Gene Expression Assay protocol in a QuantStudio 5 Real-Time PCR system (Thermo Fisher Scientific, Waltham, MA, USA). As indicated previously [20], the validation of similar reaction efficiencies in both genes was done by running serial dilution curves with four cDNA points. The 2^−ΔΔCq^ and 2^−ΔCq^ methods were used to analyze the *PADI4* expression in the GTG haplotype carriers in relation to the ACC haplotype carriers (calibrator group).

### 2.7. Isolation of Leukocytes

Leukocytes [including peripheral blood mononuclear cells (PBMC) and polymorphonuclear cells (PMN)] were obtained from RA patients (*n* = 8) and CS (*n* = 8) carrying the GTG or ACC haplotypes. The cells were separated by density gradient (Histopaque 1.119 and 1.077 Sigma-Aldrich, Misuri, USA) and were washed with phosphate buffer saline (PBS) (pH 7.2), and centrifuged at 4 °C for 10 min at 1800 revolutions per minute (rpm); then, the cell button was added in 1 mL of RPMI-1640 with 10% fetal calf serum. The number of viable cells was determined by the exclusion technique with 3% trypan blue staining; the total number of cells/mL was quantified by direct counting using a Neubauer chamber. The cells were subsequently lysed by sonication.

### 2.8. PAD4 Enzymatic Activity Analysis

The enzymatic activity of PAD4 was evaluated by the technique reported by Chikuma et al. [21], based on the conversion of the peptide Dansyl Glycyl -l- Citrulline (Dns-Gly-Cit) liberated enzymatically from the substrate, Dansyl Glycyl Arginine (Dns-Gly-Arg) (GenScript Biotech, Piscataway, NJ, USA). The generation of the Dns-Gly-Cit peptide was evaluated by HPLC and fluorescence detection. The assay medium consisted of 40 mM Tris-HCl buffer (pH 7.6), 2 mM CaCl2, 1 mM Dithiothreitol, adding the substrate Dns-Gly-Arg 20 mM. After the addition of 300 µg of protein of PBMC and PMN lysates from RA patients and CS, it was incubated at 37 °C and the reaction was stopped at different reaction times (0.5, 1, 2 and 4, and 6 h), incubating at 100 °C for 10 min. The sample was centrifuged at 4 °C for 10 min, and 20 µL was injected in triplicate for analysis by HPLC. For the analysis of PAD activity in µmol/mL/µg protein, the area of each peak corresponding to the Dns-Gly-Cit peptide at the different incubation times was integrated. The Dns-Gly-Cit was enzymatically prepared from Dns-Gly-Arg using recombinant PAD (Abcam ab170409, Cambridge, UK), and the corresponding area for each incubation time was used for the standard curve.

### 2.9. Statistical Analysis

Statistical analysis was performed by STATA v 9.2 and GraphPad Prism v 8.0 software. For the statistical analysis, *p* < 0.05 was considered significant. Variable distribution was determined using the Shapiro–Wilk normality test. The data were expressed as means and standard deviations (SD) for descriptive statistics. For the inferential statistics for two groups, a *t*-test was performed, and the ANOVA test was used for more than two groups. Variables non-normally distributed were presented in median and interquartile ranges (25–75) or median and percentiles 5–95. For the inferential statistics for two groups, the Mann–Whitney U test was performed and the Kruskal–Wallis test for more than two groups.

## 3. Results

### 3.1. Clinical and Demographic Characteristics of RA Patients

The clinical and demographic characteristics of RA patients and CS are shown in Table 1. We observed that most patients were female (87% women and 13% men). In patients with RA, increased levels of acute-phase reactants were observed, including ESR (39 mm/h) and hsCRP (24.7 mg/L). Regarding antibodies, the mean of RF was 89.3 U/mL [54% were positive for RF] and 100.2 U/mL for anti-CCP antibodies [72.7% were positive for anti-CCP antibodies]. In addition, high concentrations of anti-PADI4 antibodies (2026.9 U/mL) were identified by a quantitative method.

### 3.2. Genotype and Haplotype Frequencies of the PADI4 Gene in RA Patients and CS

The 3 SNPs of *PADI4* were in Hardy–Weinberg equilibrium in the CS group (*PADI4_89 G>A*, *p* = 0.279; *PADI4_90 T>C*, *p* = 0.305; *PADI4_92G>C)*, *p* = 1.00). The frequency distribution in RA patients and CS is shown in Table 2. We found significant differences in the frequencies of genotypes and alleles between RA patients and CS for each of the three polymorphisms analyzed. In addition, we performed haplotype analysis of the *PADI4* gene in 40 CS and 38 RA patients who were identified as homozygous for each haplotype. We observed a higher frequency of the GTG haplotype in RA patients than in CS (57.9% vs. 32.5%), while the ACC haplotype was more frequent in CS than in patients (67.5% vs. 42.1%). After performing the haplotype association analysis, a strong association of the GTG haplotype with the risk of developing RA (OR = 2.86; *p* = 0.024) was found (Table 3).

### 3.3. Allele-Specific Quantification of PADI4 mRNA Expression

**Figure 1:**
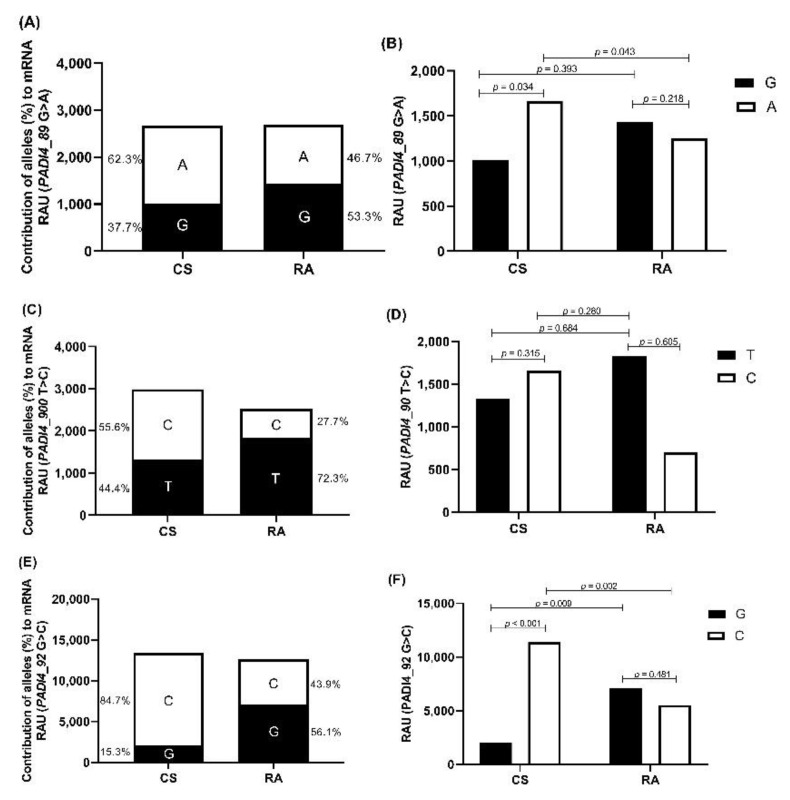
Relative contribution of the *PADI4* 89G>A, 90T>C, and 92G>C polymorphisms to *PADI4* mRNA expression. (**A**) Graphical representation of the results obtained by the ImageJ software of the allelic restriction fragments ratio indicating the relative contribution (%) of G and A alleles of the 89G>A polymorphism to *PADI4* mRNA in CS and RA patients. (**B**) Relative area units of allelic expression (G or A) of the *PADI4* 89G>A polymorphism in CS and RA patients. (**C**) Graphical representation of the results obtained by the ImageJ software of the allelic restriction fragments ratio indicating the relative contribution (%) of T and C alleles of the 90T>C polymorphism to *PADI4* mRNA in CS and RA patients. (**D**) Relative area units of allelic expression (T or C) of the *PADI4* 90T>C polymorphism in CS and RA patients. (**E**) Graphical representation of the results obtained by the ImageJ software of the allelic restriction fragments ratio indicating the relative contribution (%) of G and C alleles of the 92G>C polymorphism to *PADI4* mRNA in CS and RA patients. (**F**) Relative area units of allelic expression (G or C) of the *PADI4* 92G>C polymorphism in CS and RA patients. RAU: relative area units. *p*-Values were calculated using the Mann–Whitney U test.

Differences in the capacity of each *PADI4* allele to contribute to the mRNA expression in RA patients and CS was detected. A higher expression of mRNA carrying the 89A allele was observed in CS (62.3% 89A allele vs. 46.7% 89G allele, ratio 1.33), while in RA patients, a higher expression of the mRNA carrying the 89G allele was observed (53.3% 89G allele vs. 37.7% 89A allele, ratio 1.41) (Figure 1A). CS expressed a higher quantity of mRNA carrying the 89>A allele than RA patients (1662.80 vs. 1255.03 RAU, *p* = 0.043; Figure 1B).

Regarding the 90T>C polymorphism, CS expresses more mRNA carrying the 90C allele than the 90T allele (55.6% vs. 44.4%; ratio 1.25), while RA patients expressed higher mRNA with the 90T allele (72.3% vs. 27.7%; ratio 2.61) (Figure 1C). On the other hand, CS expressed higher mRNA carrying the 90C allele than RA patients (1660.59 vs. 699.56 RAU, *p* = 0.280; Figure 1D).

For the 92G>C polymorphism, it was observed that the CS expressed more mRNA carrying the 92C allele than the 92G allele (84.7% vs. 15.3%; ratio 5.53), while in RA patients, a higher percentage of mRNA carrying the 92G allele was observed (56.1% vs. 43.9%; ratio 1.27; Figure 1E). Furthermore, it was observed that CS expresses more the mRNA with the 92C allele than RA patients (11380.69 vs. 5536.59 RAU, *p* = 0.002; Figure 1F).

### 3.4. PADI4 Expression in RA Patients and CS

**Figure 2:**
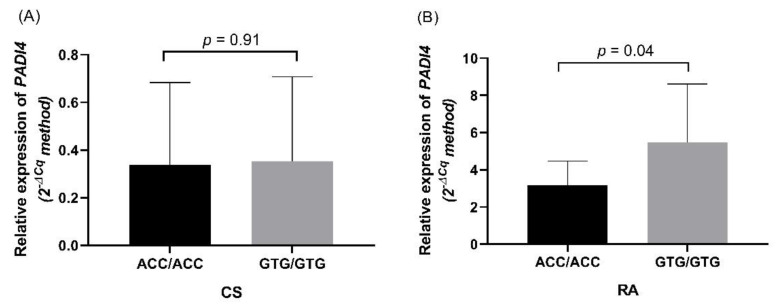
*PADI4* mRNA expression according to the ACC and GTG haplotypes of the *PADI4* gene. (**A**) *PADI4* expression according to the ACC and GTG haplotypes in CS. (**B**) *PADI4* expression according to the ACC and GTG haplotypes in RA. *P* value was calculated using the Mann–Whitney U test.

The *PADI4* mRNA expression of 20 patients and 20 CS carrying the ACC and GTG haplotypes was quantified. The mRNA expression in CS was similar in carriers of both haplotypes (*p =* 0.91, Figure 2A). In RA, homozygous for the GTG haplotype had higher *PADI4* mRNA expression than homozygous for the ACC haplotype (1.62 times; *p =* 0.04) (Figure 2B).

### 3.5. Evaluation of PAD4 Activity in CS and RA Patients

**Figure 3:**
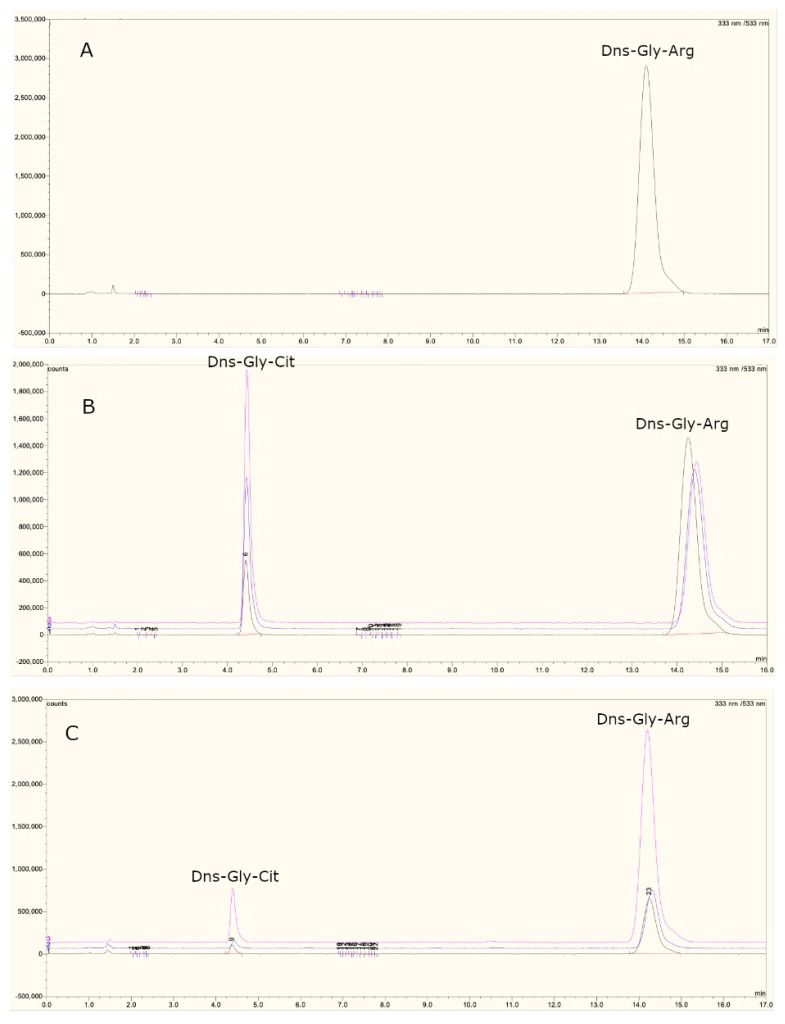
Fluorescent elution patterns of the reaction products generated by type PAD in PBMC and PMN lysates. (**A**) 20 mM Dns-Gly-Arg; (**B**) incubation with recombinant PAD enzyme in the presence of 20 mM Dns-Gly-Arg; (**C**) Incubation of 20 mM Dns-Gly-Arg with PMN and PBMC lysate from RA patients. The arrow indicates the peak corresponding to Dns-Gly-Cit.

The injection of the Dns-Gly-Arg (DGA) peptide into the chromatography equipment resulted in a chromatogram with an elution peak at minute 14.4 of the peptide without citrullination due to the peptide was in the absence of the PAD enzyme, so it was used as a reference (Figure 3A).

**Figure 4:**
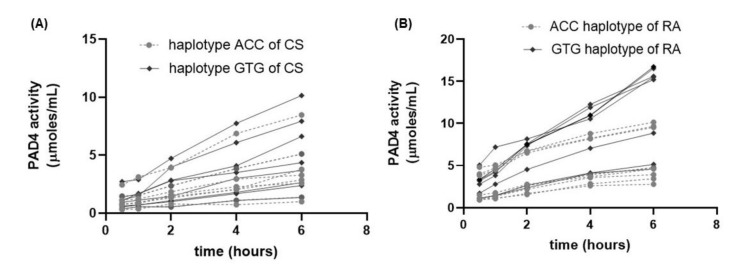
Effect of incubation time on the PAD4 activity from lysates of leukocytes from CS and RA. (**A**) Changes in µmoles/mL for each CS; ACC haplotype carriers are represented in the gray lines, while CS with the GTG haplotype are represented in the black lines. (**B**) Changes in µmoles/mL for each RA patient. RA patients carrying the ACC haplotype are represented by gray lines, while RA patients with the GTG haplotype are represented with black lines.

**Figure 5:**
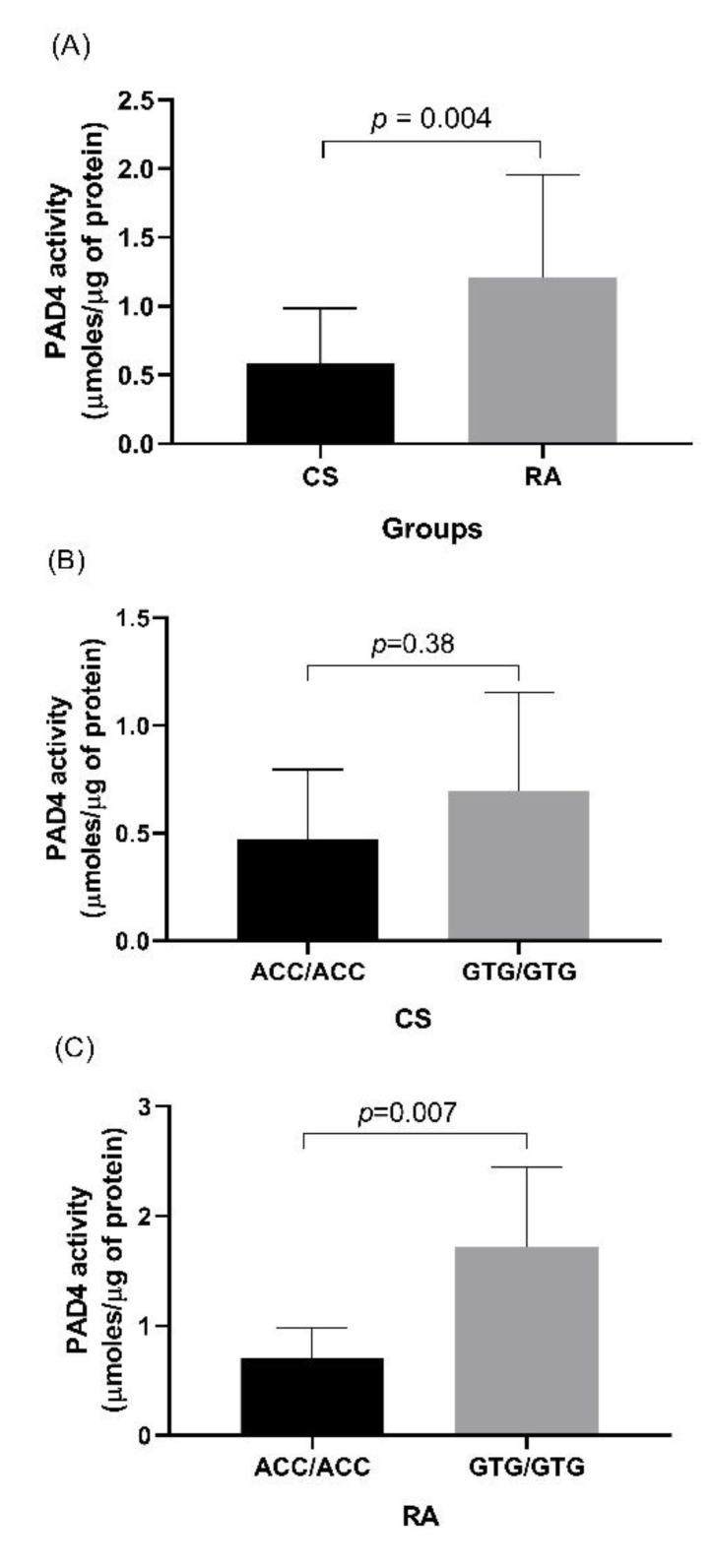
Determination of PAD4 activity in CS and RA patients. (**A**) Comparison of PAD4 activity in CS and RA carrying the ACC and GTG haplotypes. (**B**) Comparison of PAD4 activity in CS carrying the ACC and GTG haplotypes. (**C**) Comparison of PAD4 activity in RA patients carrying the ACC and GTG haplotypes. The *p* value was calculated by the Mann–Whitney U test, comparing the value of the slope obtained for each subject.

**Figure 6:**
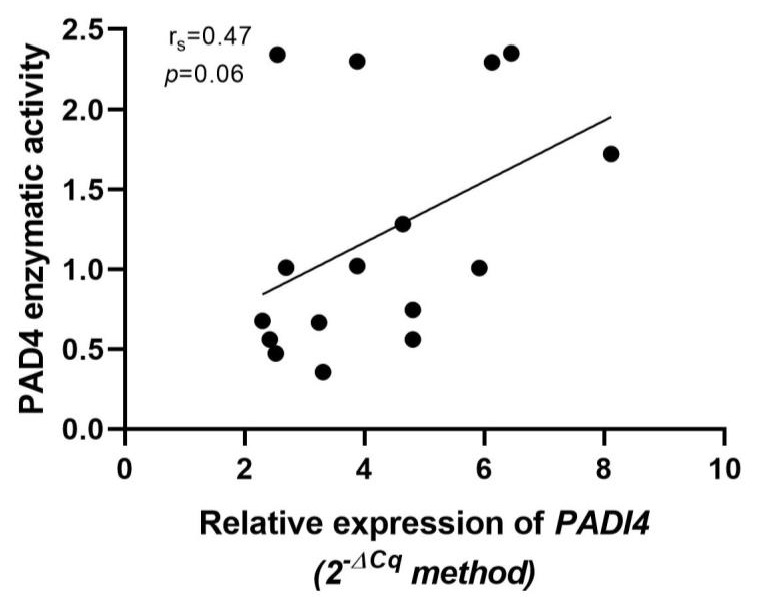
Correlation of mRNA *PADI4* expression with PAD4 enzymatic activity. The *p*-value was calculated by the Spearman correlation (rs).

In addition, it was observed that the DGA peptide in the presence of the recombinant enzyme presents an additional elution peak at minute 4.38, considered a citrullinated peptide (Figure 3B). An increase in the signal area corresponding to the citrullinated peptide was obtained during incubation from 30 min to 6 h (Figure 3C). In leukocytes, we compared the activity of PAD4 represented by the change in slope of the line obtained in a time of 0.5 to 6 h (Figure 4). RA patients presented a higher enzymatic activity of PAD4 compared to CS (Figure 5), and patients carrying the GTG haplotype presented more significant activity than those carrying the ACC haplotype (*p* = 0.007) (Figure 6).

### 3.6. Association of Relative Expression of PADI4 Gene with PAD4 Enzymatic Activity

A positive correlation between the relative expression of the *PADI4* gene and PAD4 activity was observed, but this was not statistically significant (*p* = 0.06, rs = 0.47) (Figure 6).

## 4. Discussion

RA is considered a multifactorial disease of unknown etiology with the participation of environmental and genetic factors contributing to this disease’s susceptibility, severity, and progression [22,23,24].

The *PADI4* gene plays an important role in the pathophysiology of RA because the PAD4 enzyme catalyzes the change from arginine to citrulline, generating neoantigens.

This study evaluated genotypic, allelic, and haplotypic frequencies of the *PADI4* gene, mRNA expression, and PAD4 activity in RA patients and CS. We observed that the 89GG, 90TT, and 92GG genotypes confer risk for RA in western Mexico. These results coincide with studies reported in Japan [8], Korea [11,25] and North America [12]. Furthermore, the genotypic frequencies for each polymorphism were very similar to those previously reported by our group of collaborators in the populations of western [14] and southern Mexico [13]. We also performed a haplotype analysis of the *PADI4* gene, and an association of the GTG haplotype with the risk of developing RA was found (*p =* 0.024). These results agree with those previously reported by our study group in western and southern Mexican populations [13,14] as well as others, predominantly Asian [8,25,26] [8,17,18]. Therefore, our results support the finding that these SNPs and their haplotypes are genetic risk factors for RA.

It has been described that each of the two alleles present in diploid organisms contributes equally to the production of mRNA. However, different functional alleles can lead to an unequal contribution of each allele in the total mRNA production [19]. Therefore, in the present study, the contributions of the *PADI4* alleles on the mRNA expression in samples from heterozygous RA and CS patients was analyzed by the ASTQ method. We found that CS expressed more mRNA carrying the non-susceptibility alleles 89A, 90C, and 92C, while the RA patients expressed more mRNA carrying the susceptibility alleles 89G, 90T, and 92G. According to the literature consulted, this is the first study evaluating the allelic contribution of *PADI4* SNPs on the *PADI4* mRNA expression by the ASTQ method. The results showed a differential expression of each functional *PADI4* allele in RA and CS, and that each allele can contribute unequally to the expression of the *PADI4* gene.

We also evaluated the gene expression of *PADI4*, and observed higher expression in RA patients carrying the GTG haplotypes. These results agree with a previous study carried out in this same population, where an increase in mRNA up to 3 times higher was reported in carriers of the GTG haplotype [14]. In addition, our results agree with the pioneering study of *PADI4* polymorphisms by Suzuki et al. in a Japanese population [8], in which they reported that the GTG haplotype is associated with RA risk, more stable mRNA (half-lives of 11.6 in GTG carriers vs. 2.1 min in ACC carriers), an higher levels of PAD4 protein than the ACC carriers [8]. Therefore, we support the hypothesis that the mRNA of the *PADI4* gene with the GTG haplotype accumulates in greater quantity than the mRNA with the ACC haplotype in RA, being able to form more PAD4 protein.

In view of the fact that we found an association of the GTG haplotype with the risk for RA and higher gene expression in carriers of these haplotypes, it was also of interest to evaluate the activity of PAD4 in leukocytes to determine the effect of these variants on the activity of the PAD4 enzyme; we observed higher PAD4 activity in patients carrying the GTG haplotype. The study by Hung et al. supports this finding. They showed that the exonic polymorphism of *PADI4* increases the enzymatic activity of PAD4 in Jurkat cells [27], and that the susceptible *PADI4* (GTG) haplotype for RA has a higher affinity for calcium and higher enzymatic activity than the non-susceptible haplotype. In that context, they concluded that the abnormal activation of the enzyme could be a consequence of the conformational change that the protein presents, caused by amino acid substitutions [27].

The present study suggests that the mRNA of the *PADI4* gene with the GTG haplotype is more overexpressed, leading to accumulation in greater quantity than the mRNA with the ACC haplotype, which leads to a more significant formation of PAD4 protein in leukocytes from patients with RA. It could increase the production of citrullinated peptides, with an increase in the formation of anti-CCP antibodies.

Some research groups have focused on describing the activity of the PAD4 enzyme by different methods. A recent study evaluated the capacity of serum to activate PAD4 in early RA patients. That study showed that PAD4 levels were higher in RA patients than in CS, and that the serum ability to activate PAD4 is associated with ACPA and RF positivity. Also, it reported that PAD4 levels are associated with earlier disease onset, and that these levels decrease after three months of treatment with disease-modifying antirheumatic drugs (DMARDs), indicating that anti-PAD treatment could potentially be beneficial in RA [28].

In addition, PAD activity has been reported to be associated with bone loss and osteoclast activation in mouse models [29]. In a study by Damgaard et al. [15], they detected PAD4 activity in the synovial fluid of patients with RA and observed PAD activity in 4 of the 5 patient samples found soluble PAD in all five samples analyzed. Therefore, it is suggested that citrullination may occur extracellularly within inflamed joints, contributing to a local inflammatory response. It is important to note that PAD requires high calcium levels and reducing conditions to exhibit full activity, which are provided at lower levels in samples derived from CS [3].

We analyzed PAD4 activity by HPLC, which is highly sensitive and suitable for complex samples [21]; it allowed us to investigate PAD4 activity in patient samples. PAD4 has been described in peripheral blood, CD15+ granulocytes, CD68+ monocytes, CD3+ T cells, and CD20+ B cells [30]. However, analysis of samples from RA patients in peripheral blood samples and specific tissues for citrullination is essential.

One of the limitations of this study is the sample size; therefore, it is essential to develop future studies with a larger sample size that allows us to confirm the association between the presence of the GTG haplotype and increased mRNA expression or PADI4 activity. However, the strength of the present study is that we provide a pooled analysis of the association between *PADI4* haplotypes with RA susceptibility, the genotypic and allelic contribution of *PADI4* polymorphisms to mRNA expression, and PAD4 enzymatic activity in leukocytes (PBMC and PMN). This last is important because PAD4 is mainly expressed in neutrophils and monocytes [3,31].

In conclusion, the present work provides valuable information on the role of the *PADI4* polymorphisms 89G>A, 90T>C, and 92G>C, and their haplotypes, which are associated with susceptibility to RA in our study population, and the susceptibility haplotype (GTG) is related with a greater increase in *PADI4* mRNA and more significant PAD4 activity. These findings suggest the functional impact of the GTG haplotype of *PADI4* in RA, with increased expression and enzymatic activity, promoting the generation of citrullinated proteins that could lead to increased production of autoantibodies.

## Figures and Tables

**Table 1:** Clinical and demographic characteristics of RA patients.

**Demographics**	RA (*n* = 100)
Age (years) ^a^	47 ± 14
Gender	
Female % (n) Male % (n)	87 (87) 13 (13)
Smoking	
Smokers % (n)	39 (39)
Non-smokers % (n)	61 (61)
**Clinical assessment**	
Disease evolution (years) ^b^	5 (1–10)
Early RA ≤ 1 year of evolution % (n)	29.2 (28)
Established RA > 1 year of evolution % (n)	70.8 (68)
Age of disease onset ^a^	41 ± 12
DAS28 clinical activity ^b^	5.0 (3.5–5.8)
HAQ-DI functional disability ^b^	0.6 (0.2–1)
RF (U/ mL) ^b^	89.3 (28.3–300)
Positive RF % (n)	54 (54)
Negative RF %(n)	46 (46)
Anti-CCP antibodies (U/ mL) ^b^	100.2 (2.7–185.1)
Positive anti-CCP % (n)	72.7 (72)
Negative anti-CCP % (n)	27.8 (27)
Anti-PADI4 antibodies (U/ Ml) ^b^	2026.9 (188.9–3359.6)
ESR (mm/h) ^b^	39 (23–46)
hsCRP (mg/L) ^b^	24.7 (12.3–41)

^a^ Mean ± standard deviation are shown for parametric variables; ^b^ median (25th percentile–75th percentile) for non-parametric variables; and percentages (absolute frequency) for qualitative variables. ESR, erythrocyte sedimentation rate; DAS-28, disease activity score 28; HAQ-DI, health assessment questionnaire disability index; hsCRP, high-sensitivity C-reactive protein; RA, rheumatoid arthritis; RF, rheumatoid factor. % Smoking was considered if they had a history of smoking or currently smoked.

**Table 2:** Genotypic and allelic frequency of *PADI4* polymorphisms in RA patients and CS.

Polymorphism	CS % (*n* = 100)	RA % (*n* = 100)	OR (IC 95%)	*p*
*PADI4_89* G>A				
AA ^a^	34 (34)	20 (20)	1	
GA	52 (52)	53 (53)	1.73 (0.84–3.6)	0.107
GG	14 (14)	27 (27)	3.28 (1.3–8.4)	0.005
Allele				
A ^a^	60 (120)	46.5 (93)	1	
G	40 (80)	53.5 (107)	1.72 (1.14–2.62)	0.007
*PADI4_90* T>C				
CC ^a^	33 (33)	19 (19)	1	
TC	51 (51)	55 (55)	1.51 (0.69–3.37)	0.270
TT	16 (16)	26 (26)	2.82 (1.12–7.14)	0.014
Allele				
C ^a^	58.5 (117)	46.5 (93)	1	
T	41.5 (83)	53.5 (107)	1.62 (1.07–2.46)	0.016
*PADI4_92* G>C				
CC ^a^	37 (37)	21 (21)	1	
GC	48 (48)	54 (54)	1.98 (0.97–4.07)	0.041
GG	15 (15)	25 (25)	2.94 (1.18–7.38)	0.010
Allele				
C ^a^	61 (122)	52 (104)	1	
G	39 (78)	48 (96)	1.69 (1.11–2.57)	0.009

RA, rheumatoid arthritis; CS, control subjects; OR, odds ratio; 95% CI, confidence interval. ^a^ reference category *p*-Values were calculated using logistic regression comparisons with the reference category.

**Table 3:** Distribution of the *PADI4* gene haplotypes frequencies.

Haplotype	CS % (n)	RA % (n)	OR (IC 95%)	*P*
GTG	32.5 (13)	57.9 (22)	2.86 (1.03–7.99)	0.024
ACC ^a^	67.5 (27)	42.1 (16)	1	

RA, rheumatoid arthritis; CS, control subjects; OR, odds ratio; 95% CI, confidence interval. ^a^ reference category *p*-Values were calculated by logistic regression comparisons with the reference category.

## Data Availability

The data presented in this study are not publicly available.
